# The role and regulation mechanism of Chinese traditional fitness exercises on the bone and cartilage tissue in patients with osteoporosis: A narrative review

**DOI:** 10.3389/fphys.2023.1071005

**Published:** 2023-02-28

**Authors:** Weibo Sun, Xin-An Zhang, Zhuo Wang

**Affiliations:** College of Exercise and Health, Shenyang Sport University, Shenyang, China

**Keywords:** Chinese traditional fitness exercise, osteoporosis, bone metabolism, bone mineral density, therapy

## Abstract

Osteoporosis (ops) is a systemic degenerative bone disease characterized by bone mass reduction, bone mineral density loss, bone microstructure destruction, bone fragility, and increased fracture susceptibility. Thus far, drug therapy is the main method used to prevent and treat osteoporosis. However, long-term drug treatment will inevitably lead to drug resistance and certain side effects. In response, rehabilitation treatment is generally recommended, which involves drug supplementation combined with the treatment. A Chinese traditional fitness exercise is an organic combination of sports and traditional Chinese medicine with a series of advantages such as being safe, convenient, non-toxic, and harmless. Hence, it is one of the rehabilitation methods widely used in clinical practice. By searching the CNKI, PubMed, Web of Science, Embase, Cochrane Library, and other relevant databases, our research clarifies the current situation of four kinds of Chinese traditional fitness exercises widely used in clinical practice, namely, Taijiquan, Baduanjin, Wuqinxi, and Yijin Jing. In addition, the molecular mechanism of osteoporosis is summarized in this study. Based on the research, Chinese traditional fitness exercises are expected to directly stimulate the bone through a mechanical load to improve bone density. Moderate and regular traditional Chinese fitness exercises also improve osteoporosis by regulating the endocrine system with the secretion of hormones and factors such as estrogen and irisin, which are beneficial for bone formation. Finally, the purpose of promoting bone formation, reducing bone loss, and preventing and treating osteoporosis is achieved. The various means of Chinese traditional fitness exercises have different emphases, and the effect of improving bone density differs in various parts of the body. The exercisers may choose the exercise flexibly based on their own needs. Chinese traditional fitness exercises can improve the bone density of the exercisers and relieve pain, improve balance, and regulate the psychological state. Consequently, it is worth promoting to be applied in clinical practices.

## 1 Introduction

Osteoporosis (ops) is a systemic degenerative bone disease characterized by bone mass reduction, bone density loss, bone microarchitecture destruction, bone fragility, and increased fracture susceptibility (Lane et al., 2000). Osteoporosis may occur in all genders and at any age, primarily in postmenopausal women and elderly people. In America, 10.2 million people suffer from osteoporosis and another 43.4 million are placed at an increased risk for fractures because of the low bone mass density (Arceo-Mendoza and Camacho, 2021). Meanwhile, in China, the incidence of osteoporosis is 51.6% and 10.7% in women and men above 60 years old, respectively (Ke et al., 2019). Approximately, 9 million new cases of osteoporosis are annually diagnosed worldwide. Thus, osteoporosis and its complications severely influence people’s quality of life and cause a burden to the family and society. At present, drug and rehabilitation therapies are used as common methods to prevent and treat osteoporosis. Drug treatment falls into two categories. One is using drugs to adjust the metabolism of elderly osteoporosis patients. For example, people with senile osteoporosis take supplements for the deficiency of bone calcium and certain vitamins in the body by taking a certain amount of calcium and vitamin preparations. The other is using sex hormones to stimulate bone formation to reduce bone decomposition and achieve the goal of osteoporosis treatment (Reid and Billington, 2022). The sex hormone is effective in treating osteoporosis in postmenopausal women (Gosset et al., 2021). However, long-term drug treatment will inevitably lead to drug dependence and increase the risk of side effects. Drugs used to treat osteoporosis often need to be broken down and absorbed through the digestive tract because these drugs need to be taken for a long time. Therefore, it inevitably damages the gastrointestinal mucosa. Certain osteoporosis drugs may also cause transient head pain, insomnia, anxiety, and other symptoms; female patients with osteoporosis often need to take supplement estrogen drugs such as conjugated estrogen tablets. Moreover, the long-term use of osteoporosis drugs can cause patients to become prone to endometrial hyperplasia, thereby leading to malignant tumors such as endometrial cancer. Therefore, clinical rehabilitation treatment is generally recommended as a supplement combined with drug treatment as an alternative to medical treatment. Rehabilitation is a form of comprehensive treatment, including psychotherapy, dietary guidance, physical factor therapy, and exercise therapy. Psychotherapy helps patients to overcome and accept their illnesses while improving patients’ compliance and initiative during the treatment. Scientific dietary guidance effectively helps treat patients with osteoporosis by supplementing Ca^2+^ and other trace elements related to bone formation directly and indirectly in the occurrence and development of osteoporosis. Physical factor therapy covers a wide range of therapies, including whole-body vibrations, ultrasounds, phototherapy, alternating current, direct current, magnetotherapy, cold and heat therapy, and fumigation and washing, which provides holistic treatment for osteoporosis-related aspects from different aspects. In addition, its effects include the stimulation and strengthening of muscles, bones, and ligaments; supplementing trace elements; counseling patients with negative emotions; and preventing and treating other potential complications. However, long-term comprehensive rehabilitation in the hospital may produce negative emotions for the patients and a great economic burden to their families. It will also take up more medical resources, thereby initiating the problems of the distribution of social medical resources.

Exercise therapy is very important. In 2018, the Chinese guidelines for the treatment of osteoporosis pointed out that exercise could improve body agility, strength, posture, and balance and effectively reduce the risk of falls and fractures caused by the diminishing bone mineral density. Therefore, exercise therapy is important to prevent and treat osteoporosis (Ma et al., 2019). Chinese traditional fitness exercises are aerobic exercises, guided by medical theories; it pays attention to the unity of mind, breath regulation, and movement. Moreover, it is the product of the organic integration of guiding techniques, such as qigong, martial arts, and medicine (Jahnke et al., 2010; Yeung et al., 2018; Yang et al., 2022). Regardless of the kind of traditional Chinese fitness exercise, it is based on the theories of yin and yang, zang-fu organs, qi and blood, meridians, and collaterals of traditional Chinese medicine. It takes nourishing essence, qi, and regulating spirit as the basic points of exercise, a dynamic form as the basic form of exercise, and guides the virtual, solid, dynamic, and static exercise with the theory of yin and yang. Flexion and pitching guided by opening and closing explain the harmony and unity of the form, spirit, qi, blood, surface and inner parts in sports, and fitness with the whole concept. Chinese traditional fitness exercises are clinically widely accepted by doctors, patients, and family members. They have various forms that are safe and easy to learn, create strong interest, and have almost no side effects. Taijiquan, Baduanjin, Wuqinxi, and Yijin Jing are widely used in treating osteoporosis. Taijiquan is light and soft; it combines movement and static, emphasizes the control of a breathing rhythm with consciousness, forms a linkage with action, improves the elasticity of joints and ligaments, strengthens muscle strength, promotes calcium deposition, prevents joint deformation and other diseases, and regulates the function of the viscera as a whole, which warms and takes care for the kidney and spleen. Meanwhile, Baduanjin’s exercise movement is simple and slow, and it is primarily based on an isometric contraction of muscles. It emphasizes the training of the lower limb muscle strength to improve the balancing ability of the lower limbs to prevent patients from falling and reduce the occurrence of fractures. Furthermore, training for Baduanjin increases bone stress, promotes bone formation, reduces bone resorption, and controls the progress of osteoporosis. In addition, the movements of Wuqinxi are simple and safe, and the active parts are comprehensive. Its movements involve the major muscle groups of the whole body and the joint movements of the spine, limbs, and fingers, which may improve the blood circulation of the soft tissue of the spine and other joints, help maintain the normal structure of bones and joints, prevent joint stiffness, enhance muscle strength, and delay the development of osteoporosis. Meanwhile, Yijin Jing focuses on strengthening muscles and bones. Long-term practice can improve the flexibility of muscles and ligaments, increase bone density, and also, open blood vessels to prevent and treat diseases.

A comprehensive search was performed using PubMed, CNKI, Embase, Web of Science, MEDLINE, CBM, and Cochrane Library databases. The studies were published from the database inception to January 2023. Medical subject headings (MESH), keywords, and free words were used in the retrieval strategy including “osteoporosis,” “bone losses,” “post-traumatic osteoporosis,” “senile osteoporosis,” “age-related osteoporosis,” “involutional osteoporosis,” “postmenopausal osteoporosis,” “low bone mineral density,” “Tai-ji,” “Tai Chi,” “Tai Ji Quan,” “Taiji,” “Taijiquan,” “Tai Chi Chuan,” “Baduanjin,” “Wuqinxi,” “Yijinjing,” and “Chinese exercise.” We collected all available RCTs of traditional Chinese fitness exercises on osteoporosis patients. The languages are limited to Chinese and English; the publication status is unrestricted. Exclusion criteria were as follows: 1) duplicate papers; 2) articles published in the abstract form or with incomplete data or when complete data could not be obtained after contacting the authors; and 3) no literature reports on the outcome measures. The current situation and the clinical application of four kinds of Chinese traditional fitness exercises including Taijiquan, Baduanjin, Wuqinxi, and Yijin Jing in osteoporosis are summarized in this review. The molecular mechanism is also briefly given.

## 2 The mechanism of Chinese traditional fitness exercises in improving osteoporosis

Exercise improves osteoporosis. Exercises can change the bone mass, morphology, and structure based on a mechanical load. It also affects bone metabolism by regulating the endocrine system to achieve the ultimate goal of preventing and treating osteoporosis.

The mechanical load generated by exercise acts on the bone tissue of the human body in two ways, directly stimulating the bone and indirectly affecting the bone through muscle contractions (Huang et al., 2008). The direct stimulation of mechanical loads indicates that the external force produced by exercise acts directly on the bone tissue. The bone tissue converts the changes of external forces during exercise into chemical or electrical signals. On one hand, exercise promotes the differentiation and proliferation of osteocytes to inhibit bone resorption, promote bone formation, and reduce bone mass loss. On the other hand, the bone trabeculae will be rearranged in bone remodeling during exercise, which could affect bone remodeling and change the structure and shape of the bone. Exercise improves bone strength and density, enhances the bone’s mechanical load-bearing capacity, and prevents the occurrence of osteoporotic fractures (Marques et al., 2011). Indirect stimulation of muscle contractions during exercise indicates that it could stimulate the bone tissue and produce indirect stress such as tension, shear force, and a squeezing pressure on the bone. The traction not only produces benign stimulation to the bone quality but also improves the blood supply to the bone, thereby increasing the inorganic salt deposition in the blood such as calcium and phosphorus. Bone mineral density also increases, which helps in decelerating the loss of the bone mass (Kawano et al., 2002; He et al., 2003).

**FIGURE 1:**
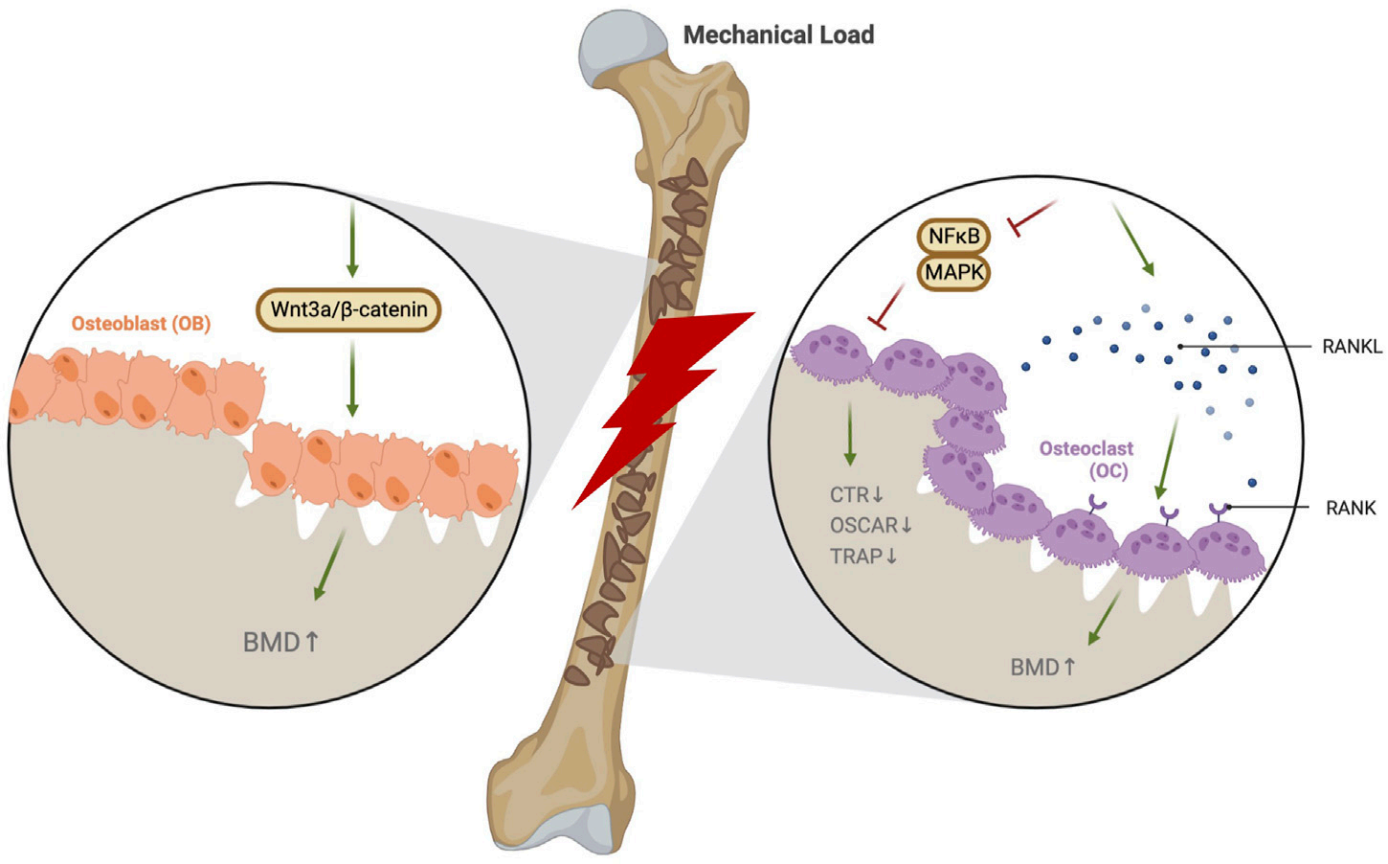
Exercise stimulates bone tissue through the mechanical load to enhance bone density.

In addition to some clinical studies showing that exercise has certain effects on osteoporosis treatment, the cellular and molecular mechanisms of exercise against osteoporosis are better understood in animal and human cell models. The stimulation of OPG/RANKL, Wnt3a/β-catenin, NF-κB, and MAPK pathways plays an important role in the progression of osteoporosis. The level of OPG increases with whole-body vibrations or treadmill stimulations in osteoporotic rats. Osteoporosis could be effectively improved by regulating the OPG/RANKL pathway by decreasing the expression of RANKL and increasing the ratio of OPG/RANKL (Tang et al., 2006; Pichler et al., 2013; Notomi et al., 2014; Yuan et al., 2016). Downhill running can promote osteoblast differentiation and osteogenic abilities to enhance bone formation and metabolism. It improves the bone morphology and structure by activating the suppressed Wnt3a/β-catenin pathway in the bones of type 2 diabetic mice (Chen et al., 2021). In addition, the LRP family proteins and Wnt/β-catenin pathway are regulated by exercise, which improves bone remodeling, bone mass recovery, and bone stiffness in rats with disuse osteoporosis (Chen et al., 2016; Jia et al., 2019). Roller exercises prevent osteoporosis by activating CD8 T cells to release IFN-γ, which exhibits the effects of inhibiting osteoclast formation and recovering bone loss through NF-κB and MAPK pathways (Shen et al., 2022) (Figure 1).

**FIGURE 2:**
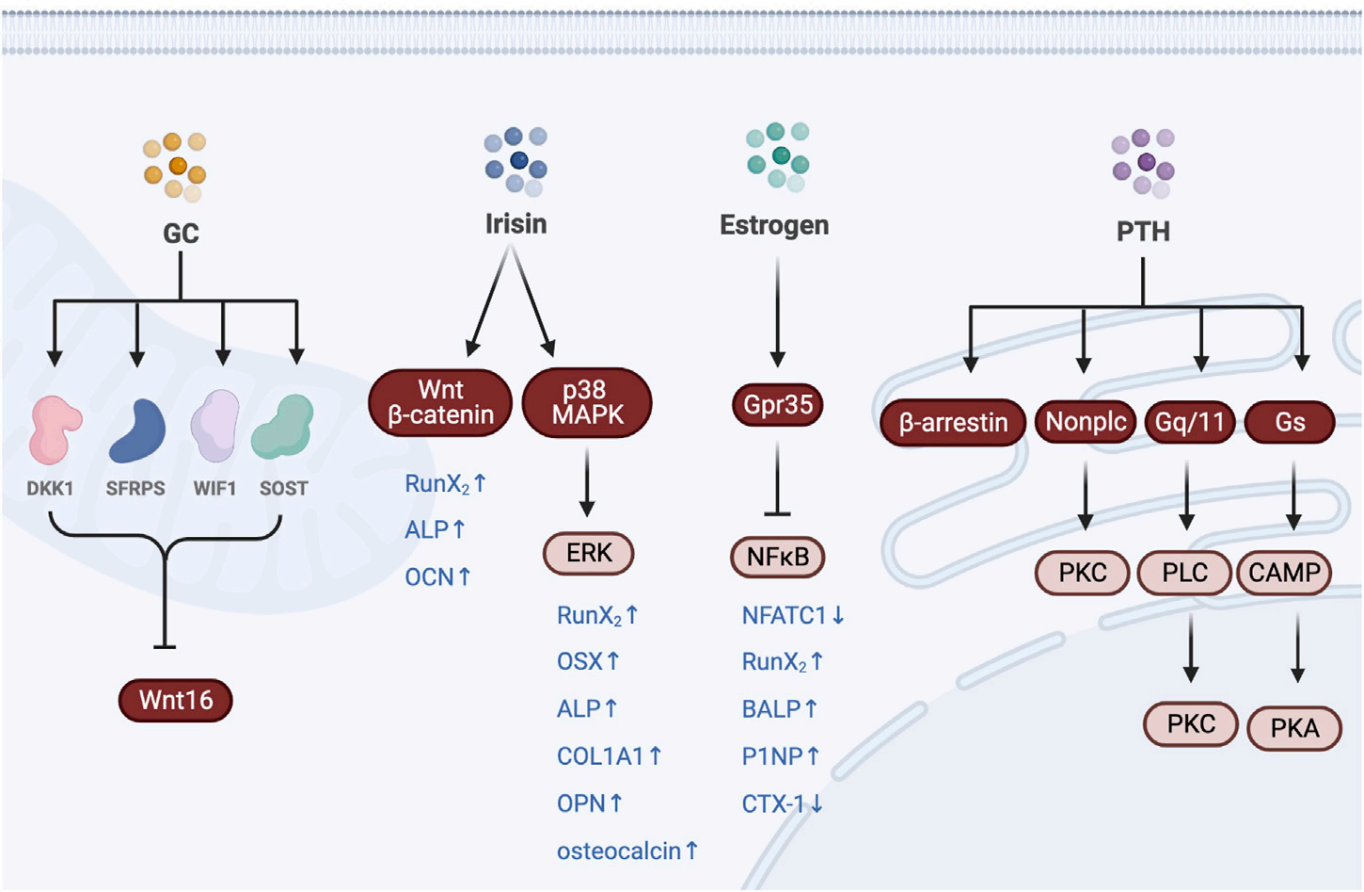
Exercise regulates bone metabolism by regulating the hormone secretion of the endocrine system.

Exercise can regulate bone metabolism by regulating hormone secretion in the endocrine system. Estrogen, muscle hormone, glucocorticoid, and parathyroid hormones participated in the process of bone metabolism (Gennari et al., 2010; Gardinier et al., 2015; Tong et al., 2019). Estrogen also promoted the proliferation and differentiation of osteoblasts while inhibiting the activity of osteoclasts and inducing osteoclast apoptosis (Robinson et al., 2009; Thouverey and Caverzasio, 2015; Lee et al., 2018; Gavali et al., 2019; Cline-Smith et al., 2020). In addition, estrogen plays a crucial role in maintaining the balance between bone resorption and bone formation to protect bone tissues. The abnormal regulation of the kynurenine pathway after estrogen deficiency may lead to the decrease of KAT expression and the concentration of KYNA of the serum in the muscles. The treadmill exercise increased the expression of the KAT enzyme in skeletal muscles while increasing the serum KYNA concentration, thereby reducing NF-κB phosphorylation by activating Gpr35. Thus, the treadmill exercise could significantly alleviate bone loss and bone microstructure destruction by inhibiting osteoclast maturation and osteoblast differentiation (Gavali et al., 2019). The iris induced by exercises can directly target osteoblasts and promote the proliferation, differentiation, and mineralization through the p38/ERK MAPK signal pathway (Qiao et al., 2016). The iris activated autophagy through the Wnt/β-catenin signal pathway and promoted osteogenic differentiation of bone marrow mesenchymal stem cells (Chen et al., 2020). Glucocorticoids negatively affect the progression of osteoporosis. Glucocorticoids inhibited the expression of Wnt16, which is an osteogenic signal to reduce the differentiation and maturation of osteoblasts by increasing the expression of Wnt pathway inhibitors such as sFRPs, WIF1, SOST, and Dickkopf-1 (DKK1) (Lane, 2019). Moreover, glucocorticoids can upregulate the expression of various factors that promote the differentiation of bone marrow mesenchymal stem cells into adipocytes, thereby decreasing the differentiation from bone marrow mesenchymal stem cells to osteoblasts (Han et al., 2019). Glucocorticoids also promoted osteoclast differentiation and maturation while extending the osteoclast life span and promoting bone resorption (Conaway et al., 2016). Exercise significantly increased blood glucocorticoid levels, which was closely related to the intensity of exercise but less related to the total amount and duration of exercise. Short-term high-intensity cycling exercises significantly increased the serum cortisol concentration in the subjects (Córdova A et al., 2019). Thus, a scientific diet and regular exercise of the appropriate intensity were strongly recommended as effective means to treat glucocorticoid-induced osteoporosis in the latest “Prevention and Treatment of Glucocorticoid-Induced Osteoporosis in Adults: Consensus Recommendations from the Belgian Bone Club” published in 2022 (Laurent et al., 2022). On the other hand, the effects of the parathyroid hormone on bone metabolism were more complex. In maintaining bone homeostasis, the parathyroid hormone played a dual role in regulating the bone metabolism primarily based on Gs-cAMP/PKA, Gq/G11/PLC/PKC, non-PLC/PKC, and β-arrestin signal pathways (Lee and Lorenzo, 2002; Yang et al., 2007; Li et al., 2017; Li et al., 2018; Zhang et al., 2018). The continuous infusion of PTH increased bone resorption and bone loss; however, intermittent PTH therapy might stimulate bone formation by reactivating the bone surface and reducing osteoblast apoptosis (Nakajima et al., 2002; Rickard et al., 2006; Friedl et al., 2007). A significant increase was observed in the blood PTH within 4 h after a 60-min load-carrying treadmill exercise, which gradually returned to pre-exercise levels within 24 h after exercise (Staab et al., 2023). Therefore, regular exercise may increase PTH intermittently and eventually stimulate bone formation (Figure 2).

## 3 Chinese traditional fitness exercises for the prevention and treatment of osteoporosis

**FIGURE 3:**
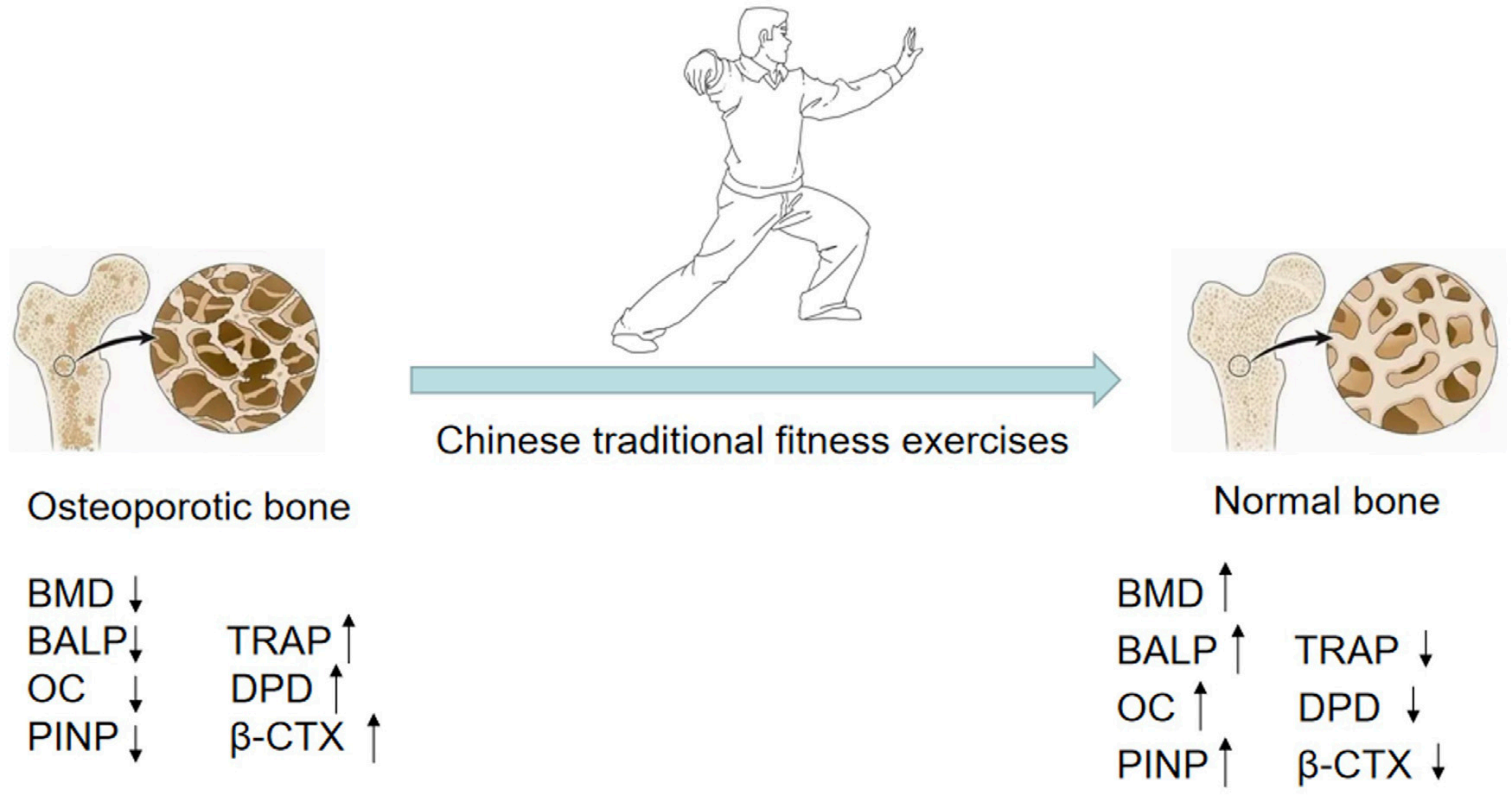
Chinese traditional fitness exercises can strengthen the bone, improve bone density, and reduce bone loss.

Researchers in different areas have confirmed various salutary functions of exercise in recent decades including improving osteoporosis. Exercise is important in increasing muscle flexibility and balance, increasing bone strength, slowing bone loss, and improving the bone structure. The guidelines for the rehabilitation of osteoporosis suggest that proper aerobic exercise functions by stimulating bone formation, regulating the endocrine system, increasing estrogen levels, promoting bone production, and preventing bone loss (Yuan and Wang, 2019). A Chinese traditional fitness exercise is a national traditional exercise with the concept of health preservation of traditional Chinese medicine combined with aerobic exercises. It is guided by “treating disease without disease” and is based on the principle of a “combination of movement and stillness, strength, and softness.” Chinese traditional fitness exercises organically combine human physical activities, breathing, and mental regulation. Long-term exercise helps reduce bone loss and relieve pain, and effectively prevents and treats osteoporosis (Figure 3).

### 3.1 Chinese traditional fitness exercises increase bone mineral density and prevent bone loss

Chinese traditional fitness exercises effectively increased the bone mineral density and reduced bone mass loss in patients with osteoporosis. In the studies on the relationship between Taijiquan and osteoporosis, Taijiquan was intervened in elderly women with osteoporosis for 9 and 12 months. The results showed that Taijiquan effectively improved the bone mineral density of the lumbar spine, hip, femur, and forearm in elderly women with osteoporosis (Wayne et al., 2012; Wu et al., 2016). In addition, the bone mineral density of the spine and femur was effectively increased after practicing Baduanjin in patients with osteoporosis (Liu et al., 2015). Hence, it further concluded that the effect of combined drug therapy with Chinese traditional fitness exercises is significant. Similarly, 6 months of the Wuqinxi and Yijin Jing exercise achieved the same effect (Jing and Zhang, 2008; Shen et al., 2013; Shen et al., 2018).

Chinese traditional fitness exercises are also beneficial to people who are not at risk of osteoporosis. A total of nine articles proved the effects of Taijiquan on the bone health of the elderly, which were detected from the spine, lumbar vertebrae, hip joint, femur, tibia, and ankle joint (Qin et al., 2002; Chan et al., 2004; Qin et al., 2005; Woo et al., 2007; Song, 2008; Zou et al., 2011; Du et al., 2014; Song et al., 2014; Song et al., 2018). Long-term Taijiquan exercise contributed to the increase of the bone mineral density of all parts of the body of the elderly in varying degrees, thereby indicating that Taijiquan played an important role in preventing osteoporosis. Zhou et al. divided 60 postmenopausal women into rope skipping, Mulan boxing, Taijiquan, and Taijiquan groups, and intervened for 10 months. It was concluded that rope skipping, Mulan boxing, and Taijiquan exercises positively affect the bone mineral density of postmenopausal women. Chinese traditional fitness exercises were effective for postmenopausal women to resist bone mass loss and promote the increase of bone mineral density. In addition, the Taijiquan hand-pushing group was comprehensive and effective in increasing the bone mass and bone mineral density (Zhou, 2004). Chen et al. conducted experiments on Baduanjin with healthy middle-aged women, for the women and Baduanjin not only maintained the bone mineral density but also decreased the level of IL-6 in the blood. Thus, it was implicated that Baduanjin effectively prevented common bone loss and was valuable in promoting and maintaining the health status of middle-aged women (Chen et al., 2006). Similarly, Wuqinxi had shown the same effect on maintaining the bone mass and bone mineral density in elderly women (Shen et al., 2013; Gu and Liu, 2021).

Nevertheless, there were certain reservations about the efficacy of Chinese traditional fitness exercises. Hui et al. conducted a randomized controlled trial on a group of middle-aged subjects. After 3 months of Taijiquan intervention, no significant change was found in the bone mineral density before and after the trial (Hui et al., 2015). However, another two studies on Taijiquan have shown that 48 weeks of Taijiquan exercise does not improve bone mineral density in perimenopausal women (Wang et al., 2015; Zhao, 2020). Li et al. believed that the short-term treatment of Wuqinxi for 6 months did not affect the lumbar BMD in patients with primary type I osteoporosis (Li et al., 2014). No significant change was observed in the bone mineral density of the femoral neck and the lumbar vertebrae in postmenopausal women after 24 weeks of a new Wuqinxi exercise but only slightly higher than that in the control group. The data suggested that the new Wuqinxi exercise may play a certain role in delaying bone loss and increasing bone mineral density (Wang et al., 2018). Meanwhile, Tsai et al. concluded that Yijin Jing played an important role in improving muscle endurance and body composition in middle-aged women, but bone strength may not be improved (Tsai et al., 2008). The aforementioned differences may be related to the different movements of traditional sports and different emphases of exercise and may also be related to the age, sex, and initial physical quality of the subjects.

### 3.2 Chinese traditional fitness exercises improve bone metabolism

Bone metabolism is the basis of ensuring the integrity of the bone composition and structure. The main form of action is the continuous absorption of old bones under the action of osteoclasts, while the continuous synthesis of the new bone is under the action of osteoblasts. A healthy bone is maintained by the dynamic balance between the absorption and reconstruction of the bone (Ma et al., 2011). Biochemical markers of bone metabolism dynamically reflect the state of bone formation and bone resorption within the organism and also reflect the changes and speed of bone matrix components. Bone alkaline phosphatase (BALP) and alkaline phosphatase (ALP) are related to bone mineralization; N-terminal propeptide (PINP) and the carboxyl-terminal propeptide (PICP) of the procollagen type I are related to a bone matrix, which is proportional to the rate of bone metabolism. In addition, the serum osteocalcin (BGP/OC) is a common biochemical marker of bone formation. The levels of pyridinoline (PYD) and deoxypyridinoline (DPD) in the urine, amino-terminal peptide (NTX), and carboxyl-terminal peptide (CTX) of type I collagen in the blood and serum acid phosphatase (TRAP) are the common markers of bone resorption. Meanwhile, calcium (Ca), phosphorus (P), and the parathyroid hormone (PTH) play regulatory roles in bone resorption and remodeling (Que and Feng, 2014). Biochemical markers of bone metabolism are greatly significant in the early detection, clinical classification, differential diagnosis, treatment monitoring, and screening of osteoporosis (Li, 2007).

Chinese traditional fitness exercises improve the level of bone metabolism in patients with osteoporosis. Wayne et al. found that 9 months of Taijiquan exercise significantly reduced the levels of serum CTX and OC in patients with postmenopausal osteoporosis, thereby suggesting that Taijiquan might reduce bone resorption and improve bone metabolism (Wayne et al., 2012). Similarly, the increase in the levels of serum ALP, Ca, and P and the decrease in the level of DPD/Cr were more effective with a 6-month Baduanjin exercise routine combined with drug intervention compared to simple drug therapy for patients with diabetes mellitus complicated with osteoporosis. In addition, Wuqinxi significantly reduced the levels of serum ALP and BGP in postmenopausal women with osteoporosis and decreased the level of PYD in urine that was equivalent to the effect of a calcium supplement alone (Shen et al., 2014). Li et al. also found that calcium supplements lasting for 9 months combined with modified Wuqinxi were effective in reducing serum PINP and S-CTX levels (Li et al., 2014).

Moreover, conventional Chinese traditional fitness exercise interventions also protected those at risk of osteoporosis. Shen et al. conducted 24 weeks of Taijiquan and resistance training in older people with the highest risk of osteoporosis. The results showed that Taijiquan and resistance training exhibited certain effects against osteoporosis, while the effects were different at different times. The level of the serum BALP in both TC and RT groups increased, but with a 6-week exercise, the level in the TC group was higher than that in the RT group. The serum PTH level in the TC group was significantly higher than that in the RT group with 12 weeks of exercise. The urine Ca^2+^ level in the TC group decreased relatively to the baseline, while that in the RT group increased after 24 weeks of exercise (Shen et al., 2007). It was implicated that the Taijiquan exercise contributed to the regulation of bone metabolism and reducing bone resorption, which showed better effects than resistance exercise. The Taijiquan intervention combined with certain active components in postmenopausal women helped effectively increase the levels of serum BAP and PTH such as green tea polyphenols (Shen et al., 2012).

### 3.3 Chinese traditional fitness exercises relieve pain

Chinese traditional fitness exercises are simple and slow. The unique movement of “lifting tendons and pulling bones” helps people’s muscles, tendons, and bones to be lifted gradually. The movements of pulling, pressing, and stretching are gentler to achieve the purpose of promoting cardiovascular circulation and regulating the metabolism. Chinese traditional fitness exercises play a significant role in relieving the bone, joint, and low back pain caused by bone loss. Meanwhile, for postmenopausal women and elderly patients with osteoporosis, regular Baduanjin exercise also effectively relieved pain. The back pain score of the Baduanjin exercise group was significantly lower than that of the control group and lasted until a subsequent follow-up (Liu et al., 2015). Similar results were also observed for Wuqinxi and Yijin Jing. Both traditional and improved Wuqinxi reduced the pain score of patients with postmenopausal osteoporosis (Jing and Zhang, 2008; Shen et al., 2013; Li et al., 2014; Shen et al., 2014) (Table 1).

**TABLE 1:** Different Chinese traditional fitness exercise types on the human OP.

Participant	Duration	Study group (*n*)	Outcome measures			Reference
			BMD	Bone metabolism	Pain	
Elderly women	48 weeks	Taijiquan (28), brisk walking (29), and square (32)	EG: lumbar spine↑ femoral neck↑			Song et al. (2018)
Menopausal women	10 months	Mulan boxing (12), Taijiquan (12), skipping (12), Taiji pushing hand (12), and control (12)	EG: lumbar spine↑ ulna↑ radius↑			Zhou (2004)
Perimenopausal women		Taijiquan (20) and control (20)	EG: lumbar spine↑ femoral neck↑	EG: BGP↓ ALP↓	EG: VAS↓	Song (2008)
Perimenopausal women	6 months	Taijiquan softball (15) and control (15)	EG: spine↑ the whole body; ↔ upper limb↔ pelvis↔	EG: Ca↔ P↔ Mg↔ ALP↔		Du et al. (2014)
Perimenopausal women	48 weeks	Taijiquan (36) and control (38)	EG: lumbar spine↔ femoral neck↔ large rotor↔ Ward’s triangle↔			Zhao (2020)
Menopausal women		Taijiquan (31) and control (28)	EG: lumbar spine↑ femoral neck↑ Ward’s triangle↑		EG: balance↑	Zou et al. (2011)
Community living elderly people	12 months	Taijiquan (58), resistance exercise (59), and control (59)	EG: lumbar spine↔ total hip↑			Woo et al. (2007)
Postmenopausal women	12 months	Taijiquan (65) and control (67)	EG: lumbar spine↔ proximal femur↔ distal tibia↑			Chan et al. (2004)
Postmenopausal women		Taijiquan (48) and control (51)	EG: lumbar spine↑ proximal femur↑ Ward’s triangle↑			Qin et al. (2005)
Elderly females	12 months	Taijiquan (35), dance (35), and walking (35)	EG: calcaneus↑		EG: balance↑	Song et al. (2014)
Postmenopausal women	12 months	Taijiquan (17) and control (17)	EG: lumbar spine↑ femoral neck↑ distal tibia↑ ultradistal tibia↑			Qin et al. (2002)
Postmenopausal women	12 months	Traditional Taijiquan (40), simplified Taijiquan (40), and control (39)	EG: lumbar spine↑ femoral neck↑ Ward’s triangle↑			Wang et al. (2015)
Adults	12 weeks	Taijiquan (129), walking (121), and control (124)	EG: lumbar spine↑ total hip↑			Hui et al. (2015)
Postmenopausal osteogenic women	9 months	Usual care(43) and Taijiquan (43)	EG: lumbar spine↑ Total hip↑ femoral neck↑	EG: CTX↓ OSC↓	EG: SF-36↔ MENQOL↔ PAR↔ balance↑	Wayne et al. (2012)
Elderly people	24 weeks	Taijiquan (14) and resistance training (14)		EG: PTH↑ urinary calcium↓ PYD ↔ BAP↑		Shen et al. (2007)
Postmenopausal women	6 months	Placebo + Taijiquan (37) and placebo (37)		EG: Ca↔ PTH↑ urinary calcium↔ P↔ BAP↑ BAP/TRAP↑		Shen et al. (2012)
Postmenopausal osteogenic women	6 months	Placebo + Taijiquan (37) and placebo (37)		EG: 8-OHdG↓ GTP↑		Qian et al. (2012)
Osteoporosis in elderly females	24 weeks	Taijiquan (30) and control (31)	EG: anklebone↑		EG: SF-36↑ balance↑	Chyu et al. (2010)
Osteoporosis in elderly females	6 months	Taijiquan (22) and control (22)			EG: SF-36↑ balance↑	Alp et al. (2009)
Community-dwelling females	12 weeks	Taijiquan (31)			EG: balance↑	Murphy and Singh (2008)
Osteoporosis in elderly males	18 weeks	Taijiquan (25) and control (24)			EG: balance↑	Maciaszek et al. (2007)
Healthy middle-aged females	12 weeks	Baduanjin (44) control (43)	EG: BMD↑	EG: IL-6↓		Chen et al. (2006)
Postmenopausal osteogenic females	12 months	Control (42), Ca (45), Baduanjin (48), and Baduanjin + Ca (49)	EG: lumbar spine↑ femoral neck↑		EG: VAS↓ balance↑	Liu et al. (2015)
Osteoporosis in elderly people	6 months	Baduanjin + Caltrate D (44) and Caltrate D (44)			EG: balance↑	Li et al. (2019)
Elderly females	24 weeks	Wuqinxi (36) and control (35)	EG: lumbar spine↔ femoral neck↔		EG: balance↑	Wang et al. (2018)
Patients with primary type I osteoporosis	6 months	Wuqinxi (30) and control (30)	EG: lumbar spine↔	EG: PINP↓ S-CTX↓	EG: VAS↓	Li et al. (2014)
Elderly people	2 weeks	Wuqinxi (75) and drug therapy (70)	EG: lumbar spine↑ femoral neck↔ large rotor↔ Ward’s triangle↔			Gu and Liu (2021)
Elderly patients with osteoporosis	6 months	Wuqinxi (100) and drug therapy (100)		EG: Ca↔ P↔ BGP↔ ALP↔ PYD↔	EG: VAS↓	Shen et al. (2014)
Osteoporosis	6 months	Yijin Jing (30) and control (30)	EG: lumbar spine↑ femoral neck↑		EG: VAS↓	Jing and Zhang (2008)
Healthy middle-aged females	8 weeks	Yijin Jing (37) and control (34)	EG: BMD↑			Tsai et al. (2008)
Old patients with primary osteoporosis	6 months	Yijin Jing (40) and drug therapy (40)	EG: proximal femur↔		EG: VAS↓ ADL↑	Shen et al. (2018)

Chinese traditional fitness exercises play a significant role in improving and preventing osteoporosis. Nevertheless, there are different types, intensities, and durations of Chinese traditional fitness exercises that may have different effects. Therefore, the exercise should be carried out in combination with the actual situation of various populations. People should carry out the appropriate intensity of pertinent targeted exercises under the guidance of professionals. Generally, the amount of exercise must reach 70%–80% of the maximum heart rate. The more commonly used calculation method is as follows: the optimal heart rate range = (220-age) × (70%–80%). Meanwhile, the exercise frequency is 3–5 times per week, and the time should be controlled between 20 and 60 min each time. It is appropriate not to feel tired the next day. In terms of the duration of exercise, it takes more than 1 year to physiologically increase bone mass. However, strict attention must be paid to safety and preventing falls during the exercise to avoid the occurrence of osteoporotic fractures.

## 4 Conclusion and prospect

Aerobic exercise is the most appropriate training for people with osteoporosis because of their fragile bones. As one of the manifestations of aerobic exercise, Chinese traditional fitness exercises have significant advantages in preventing and treating osteoporosis. In addition, Chinese traditional fitness exercises increase bone mass, decelerate bone loss, and improve bone metabolism, and play a significant role in improving balance, reducing fall risks, relieving pain, and improving the quality of life. Chinese traditional fitness sports need low skill requirements and have a higher safety index than ordinary aerobic exercises. It is rich in various movements, which meet the different exercise needs of different people. Chinese traditional fitness sports bring enormous interest and greatly improve patients’ compliance. However, there are certain limitations to Chinese traditional fitness exercises. The effects are generally slow, while the courses of treatment are relatively long. In addition, there is an upper limit for the improvement of bone mass. When the bone mass increases up to a certain level, continuous exercise may still slow down the rate of bone loss, but it will not continue to increase bone mineral density (Benedetti et al., 2018). However, certain skills are necessary for some of the movements of Chinese traditional fitness exercises. If unqualified or wrong exercises are carried out for a long time, they may reduce the curative effects and even cause injury.

The current studies mostly focused on the clinical effects such as the bone mineral density, bone metabolism index, and pain, but there was no way to verify them by special animal experiments. There is a lack of discussion of the mechanism at the present stage. In addition, the sample size of most clinical trials was relatively small, and some trials did not strictly follow the random and double-blinded principles. Thus, the problem of the short intervention time and lack of long-term follow-up reports remains. Moreover, there is no set of unified guidelines on how to choose the appropriate exercise therapy, and there is no unified standard for the time and frequency of each exercise. Therefore, it is essential to formulate a standardized, reasonable, and scientific exercise prescription, and thus, this will be our next research direction.

Our work was performed concerning the effects of Chinese traditional fitness exercises against osteoporosis. This review may contribute to the development of more targeted methods to improve and prevent osteoporosis to provide theoretical references. Herein, molecular mechanisms of exercises in improving osteoporosis are also summarized, which can further aid the development of rational therapies based on Chinese traditional fitness exercises. We believe that this review may lay the foundation for the development of appropriate treatments to prevent and treat osteoporosis.
